# Multi-layered Gag-specific immunodominant responses contribute to improved viral control in the CRF01_AE subtype of HIV-1-infected MSM subjects

**DOI:** 10.1186/s12865-016-0166-8

**Published:** 2016-08-30

**Authors:** Fanming Jiang, Xiaoxu Han, Hui Zhang, Bin Zhao, Minghui An, Junjie Xu, Zhenxing Chu, Tao Dong, Hong Shang

**Affiliations:** 1 Key Laboratory of AIDS Immunology of National Health and Family Planning Commission, Department of Laboratory Medicine, The First Affiliated Hospital, China Medical University, Shenyang, China; 2 Collaborative Innovation Center for Diagnosis and Treatment of Infectious Diseases, Hangzhou, China; 3 Medical Research Council Human Immunology Unit, Weatherall Institute of Molecular Medicine, John Radcliffe Hospital, Oxford University, Oxford, OX3 9DS United Kingdom

**Keywords:** HIV-1, CTL, CRF01_AE, Gag, Immunodominant

## Abstract

**Background:**

The purpose of this study was to characterize specific cytotoxic T-cell (CTL) responses in men who have sex with men (MSM) subjects infected with the human immunodeficiency virus type 1 (HIV-1) CRF01_AE subtype during the first year of infection and impacts on viral control and evolution.

**Results:**

Fifteen HIV-1 primary infected cases were recruited from Liaoning MSM prospective cohort. CTL responses to Gag, Pol and Nef proteins at 3 month and 1 year post infection were detected with Gamma interferon enzyme-linked immunospot (ELISPOT) assay using optimized consensus overlapping peptides, as well as the viral quasispecies sequences from the synchronous plasma. Gag and Nef proteins were the main targets of CTL responses during the first year of HIV-1 infection, and this was evident from the data after adjusting for the length of amino acids by dividing the amino acids number of the corresponding protein and multiplying by 100. Additionally, relative magnitudes of Gag at both 3 months and 1 year post infection were significantly negatively correlated with the viral set point (*p* = 0.002, *r* = −0.726; *p* = 0.025, *r* = −0.574). While the relative magnitude of Nef at 1 year post infection were significantly positively correlated with viral set point (*p* = 0.004, *r* = 0.697). Subjects with multi-layered Gag immunodominant responses during the first year of infection had significantly lower viral set points than subjects without such responses (*p* = 0.002).

**Conclusion:**

Multi-layered Gag immunodominant responses during the first year of infection were correlated with viral control, which provides a theoretical basis for vaccine design targeting MSM subjects with the CRF01_AE subtype.

**Electronic supplementary material:**

The online version of this article (doi:10.1186/s12865-016-0166-8) contains supplementary material, which is available to authorized users.

## Background

Acute human immunodeficiency virus type 1 (HIV-1) specific cytotoxic T-cell (CTL) responses play a pivotal role in controlling viral infection [1, 2]. The first appearance of CTL response coincides with [3, 4] and makes a contribution to the decline of peak viremia in acute infection [5, 6]. Acute CTL response is a critical determinant of the viral set point [7–9], which has been proven to be a strong predictor of the rate of disease progression [10]. Under the pressure of CTL response, HIV mutates to escape from recognition [6, 11]. Escape mutation may revert to the wild type upon viral transmission into a human leukocyte antigen (HLA) mismatched individual [12]. Although compelling evidence suggests that HIV-1-specific CD8^+^ T-cells play an important role in viral control, antiviral efficacy is heterogeneous. First, CTLs have different antiviral effectiveness when they target different viral proteins [13–16]. Many studies have shown that targeting the Gag protein, but not the Env or Nef proteins, is associated with improved disease control [17, 18]. Second, certain HLA types are associated with different outcomes of HIV infection [19, 20]. For example, expression of HLA-*B57, B*5801 and B*27 is associated with successful HIV control [12, 17]. In contrast, expression of HLA-B*5802 and B*3502/03 is associated with failure to control HIV [20, 21]. These HLA associations suggest that the specificity of HLA-restricted CTL responses is linked to the rate of disease progression. Third, the characteristics and effectiveness of CTL responses may be discordant among populations with different HLA distributions and viral subtypes [22, 23]. For example, CTL responses restricted by HLA-B*1503, which was shown to be rare in a B subtype cohort but common in a C subtype cohort, were associated with a lower viral load in the B subtype cohort. However, such responses were not associated with a lower viral load in the C subtype cohort [22].

HIV-1-specific CTL responses present clear immunodominance patterns during early HIV-1 infection, with a small number of epitopes being targeted in a distinct hierarchical order [6, 11, 24–26]. The immunodominance patterns of T-cell responses are determined by multiple factors, including kinetics of viral protein expression, virus sequence, HLA distribution, binding avidity of peptides to the HLA molecule and T-cell receptor repertoire [27, 28]. Immunodominant responses can be defined as common reactive epitopes targeted by individuals with a specific HLA distribution at the population level [26, 29] and the strongest response in a subject who has developed more than one varying response, which can be highly variable in different subjects and may change over time largely due to HIV-1 sequence variation [6, 11]. Immunodominance at the population level has been widely investigated. Studies have previously identified several immunodominant responses as being associated with improved HIV control [9, 26]. Immunodominance at the individual level was found to be a major determinant of epitope escape [11], although information regarding the relationship between immunodominant responses at the individual level and virus control are lacking [6, 11, 30].

In China, the number of people living with HIV has increased in recent years [31], and the incidence of transmission through men who have sex with men (MSM) has shown a marked uptrend, increasing from 2.5% in 2006 to 25.8% in 2014 [31]. CRF01_AE has become the predominant genotype among MSM in China [32–34]. Two CRF01_AE lineages have been identified in this population, with cluster I spread widely across China and cluster II observed mainly as an epidemic in northern China [32]. By far, the information on early CTL responses regarding the CRF01_AE subtype in Chinese MSM subjects is limited. Data from CTL studies in early infection have primarily been acquired from Caucasian or African populations infected with B or C subtype viruses [6, 17, 26]. The HLA distributions among Caucasian and African populations differed from Chinese populations [35], thus, it is urgent to clarify the characteristics of CTL responses and their relationship with virus control.

In this study, we aimed to elucidate the characteristics of CTL responses to CRF01_AE subtype virus during the early stage of infection and explore the protective CTL responses correlative with viral control. We designed a set of overlapping peptides to HIV-1 Gag, Pol and Nef proteins based on the previously identified CRF01_AE cluster II consensus sequence [32]. The CTL responses at 3 months and 1 year post infection were detected longitudinal with 15 primary CRF01_AE cluster II HIV-1 infected MSM subjects. The viral quasispecies sequences from the synchronous plasma were also analyzed.

## Methods

### Study population

In this study, 15 subjects with the CRF01_AE subtype cluster II of primary HIV-1 infection were recruited from a large-scale, high risk MSM cohort in Liaoning, China. Blood samples and epidemiological information were acquired every 10 weeks. HIV antibody screening was tested with third-generation ELISA and rapid test, and validation was performed with western blot assay. Antibody negative samples were tested with HIV nucleic acid. Homosexual transmission modes were confirmed by epidemiological investigation for all subjects. The date of infection was estimated as 14 days prior to RNA-positive and antibody-negative results (9 subjects) or the middle time between the last antibody-negative test and the first antibody-positive test (6 subjects). The average age of the 15 subjects was 35 ± 14 years old. Peripheral blood mononuclear cells (PBMCs) from the 15 subjects were separated by Ficoll-Paque^TM^ Plus (GE Healthcare Bio-Sciences AB, Sweden) density gradient centrifugation and cryopreserved in liquid nitrogen. The viral set point was defined as the average viral load (at least 3 time points) from 120 days to 1 year post infection, and the mean viral set point of the 15 subjects was 4.24 log_10_copies/ml (Table 1). The CD4^+^ T-cell counts of all the subjects were more than 200 cells/μl during the first year of infection and all the subjects were anti-retroviral naïve during the first year of infection. The signed written informed consents were got from all subjects for blood collection and the following study. The First Hospital of China Medical University Ethics Committee approved this study (2011–36).

**Table 1:** Subject demographics and clinical data

PID	Subtypes^a^	Median CD4 counts^b^	Set point^c^	HLA types		
				HLA-A	HLA-B	HLA-C
Subject 1	CRF01_AE	671	2.84	01,33	07,35	07,12
Subject 2	CRF01_AE	507	2.95	02,29	27,40	02,03
Subject 3	CRF01_AE	578	3.53	03,24	15,49	07,08
Subject 4	CRF01_AE	657	3.58	02,24	13,40	03,08
Subject 5	CRF01_AE	670	3.75	02,32	13,44	03,04
Subject 6	CRF01_AE	557	4.29	01,02	15,57	06,06
Subject 7	CRF01_AE	498	4.30	02,26	38,48	08,12
Subject 8	CRF01_AE	407	4.43	02,11	13,13	02,03
Subject 9	CRF01_AE	614	4.49	02,80	15,15	03,03
Subject 10	CRF01_AE	416	4.50	02,30	13,46	01,06
Subject 11	CRF01_AE	254	4.61	02,02	15,58	03,03
Subject 12	CRF01_AE	695	4.62	02,11	40,46	01,08
Subject 13	CRF01_AE	280	5.08	02,30	13,13	06,07
Subject 14	CRF01_AE	440	5.20	02,11	15,40	03,07
Subject 15	CRF01_AE	664	5.38	11,11	35,54	01,01

^a^Subtypes were all CRF01_AE cluster II

^b^Median CD4 counts were calculated from at least 3 time points from 3 months to 1 year post infection

^c^Set point was the average viral load from 120 days to 1 year post infection (at least 3 time points)

### CD4^+^ T-cell counts, viral load measurement, and HLA typing

CD4^+^ T-cell counts were determined using a FACS Calibur flow cytometer (Becton-Dickinson, USA). Plasma HIV-1 RNA levels were measured with the COBAS AmpliPrep/COBAS TaqMan HIV-1 test (Roche, Germany). HLA typing was performed using the polymerase chain reaction sequence-specified primer (PCR-SSP) method, with two-digit specificities (One Lambda, USA).

### Synthetic peptides

In order to detect the CTL responses as more as possible, a set of cluster specific 18-mer peptides with 10 overlapping amino acids spanning Gag, Pol and Nef genes were synthesized by Sigma-Aldrich (U.S.). This set of peptides was designed based on the consensus sequence of 50 near full length sequences from acute CRF01_AE cluster II infected subjects which came from a HIV seronegative prospective MSM cohort,with definite laboratory and epidemiology evidences for acute infection. A total of 209 overlapping peptides (OLPs) were synthesized; screening was performed using a 14 × 16 matrix design in which the principle is to mix the Gag, Pol and Nef peptides in the second dimension rather than the first dimension (Additional file 1: Table S1). Peptides with any response in a given matrix allowed identification of the common peptide represented in the two corresponding pools. The identity of each peptide was confirmed using PBMCs from the same time point. We also synthesized optimal epitopes restricted by common HLA-I alleles (A*0201, A*11, A*24, B*13, B*27, B*40, B*51) in the Chinese population [35] (Additional file 2: Table S2). Optimal epitopes were synthesized according to sequences from CRF01_AE subtype cluster II.

### IFN-γ ELISPOT assay

IFN-γ enzyme-linked immunospot (ELISPOT) assays were performed as previously described [36]. In brief, PBMCs were plated in 96-well plates that were precoated with anti-IFN-γ monoclonal antibody at 100,000 cells per well with peptides at a final concentration of 5 μg/ml for pooled peptides, single peptides, and optimal epitopes (BD^TM^ ELISPOT, USA). Phytohemagglutinin (PHA) served as a positive control at a concentration of 10 μg/ml, and medium alone was the negative control. Spots were counted using the ImmunoSpot® Analyzer (Cellular Technology Ltd, USA), and the number of specific T-cells was counted by subtracting the mean negative control values. Responses were considered positive if the activity was at least three times greater than the mean number of spot-forming cells (SFCs) in the negative control as well as >50 SFCs per 10^6^ PBMCs.

### Gag sequencing

Viral RNA was extracted from plasma samples obtained from MSM subjects with the QIAamp Viral RNA Mini-kit (Qiagen, UK). The Gag gene was amplified using the SuperScript Polymerase One-Step RT-PCR System (Takara, Dalian, China), followed by a second round of PCR with Gag-specific primers [36]. PCR products were gel purified with the QIAquick gel extraction kit (Qiagen, UK) and cloned with a TOPO TA cloning kit (Invitrogen, USA). The cloning PCR fragments were then sequenced with Sanger sequencing method using ABI 3730 DNA analyzer by Huada Genomics Company (China). A sequencer program was used to assemble and edit individual sequence fragments.

### Statistical analysis

Data analysis and graphical presentation were performed with IBM SPSS version 20.0 software and GraphPad Prism version 5.0 software. Statistical analysis of significance was performed using either the Wilcoxon signed-rank test or Friedmen’s two-way analysis of variance by ranks followed by pairwise multiple comparisons for comparing characteristics of HIV-1-specific CTL responses and using the Mann-Whitney test for comparing the viral set points between different groups. Spearman rank correlation was used to assess relationships between viral set points and HIV-specific T-cell responses. A p value less than 0.05 was considered statistically significant.

## Results

### Characteristics of HIV-1-specific CTL responses during the first year of infection

**Fig. 1:**
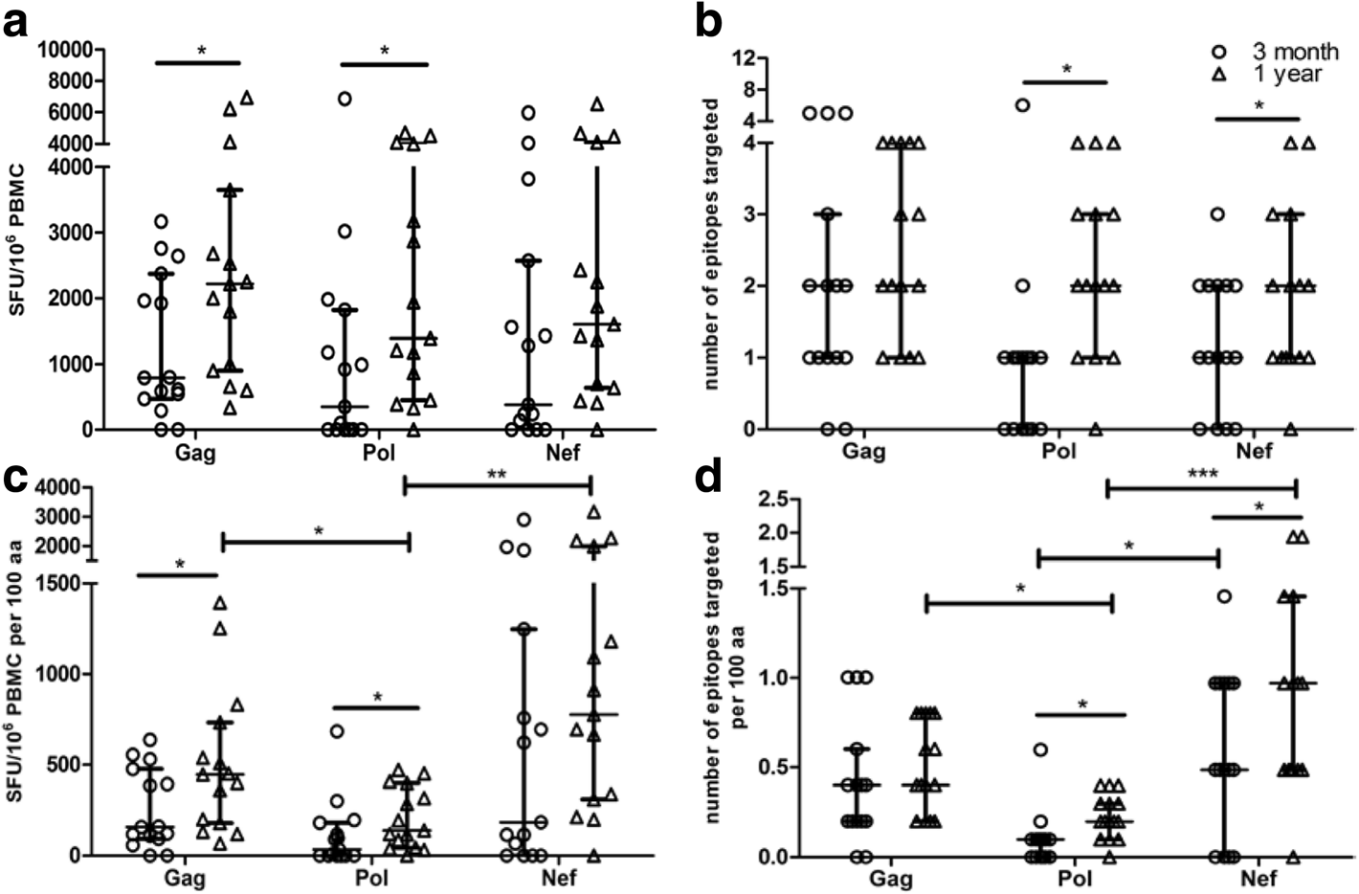
Characteristics of HIV-specific CTL responses at 3 months and 1 year post infection. The magnitude (**a**) and breadth (**b**) of Gag-, Pol- and Nef-specific CTL responses at 3 months and 1 year post infection. Adjusted magnitude (**c**) and adjusted breadth (**d**) of Gag-, Pol- and Nef-specific CTL responses at 3 months and 1 year post infection. Adjusted magnitude and adjusted breadth were calculated by dividing the amino acids number of the corresponding protein and multiplying by 100. Circles depict Gag-, Pol- and Nef-specific CTL responses and adjusted responses at 3 months post infection; triangles depict Gag-, Pol- and Nef-specific CTL responses and adjusted responses at 1 year post infection. * *p* < 0.05, ** *p* < 0.01, *** *p* < 0.001

To clarify the characteristics of CTL responses during the first year of infection, Gag-, Pol- and Nef-specific CTL responses were evaluated longitudinally in 15 MSM subjects with the HIV-1 CRF01_AE subtype at 3 months and 1 year post infection. There were no significant differences at either time point among Gag-, Pol- and Nef-specific CTL responses in terms of magnitude and breadth (*p* > 0.05). The magnitude and breadth of Gag, Pol and Nef responses increased over time, with significant differences in the magnitude of Gag and Pol (*p* < 0.05 for both) and breadth of Pol and Nef (*p* < 0.05 for both) (Fig. 1a, b).

We adjusted for the length of amino acids by dividing the amino acids number of the corresponding protein and multiplying by 100, and found that Gag and Nef responses were the mainly targets during the first year of infection. The adjusted breadth of Gag was significantly broader than that of Pol at 1 year post infection (*p* < 0.05, Fig. 1d). Similarly, the adjusted breadth of Nef was significantly broader than that of Pol at 3 months and 1 year post infection (*p* < 0.05 and *p* < 0.001, respectively, Fig. 1d). In addition, the adjusted magnitude of Gag and Nef were significantly higher than that of Pol at 1 year post infection respectively (*p* < 0.05 and *p* < 0.01, respectively, Fig. 1c). There were no significant differences among Gag, Pol and Nef in adjusted magnitude at 3 months post infection. Different protein regions presenting different T-cell responses may be associated with variations in viral control.

### Relative magnitude of Gag-specific responses showed a significantly inverse association with viral set point during the first year of infection

**Fig. 2:**
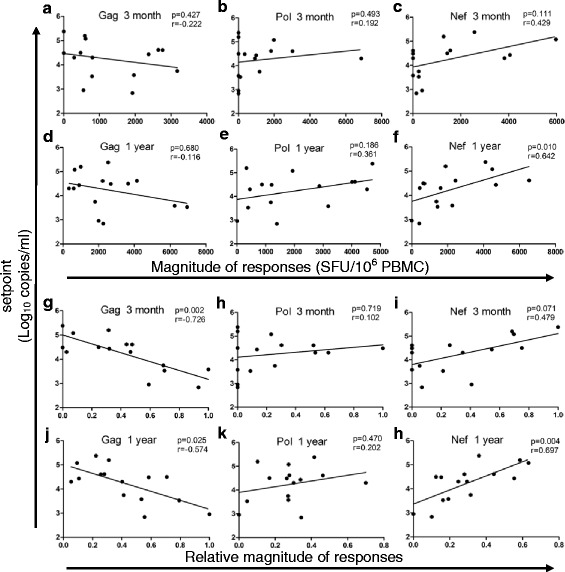
Association between viral set point and HIV-1-specific CD8^+^ T-cell responses. (**a**–**c**) Spearman correlation between magnitude of HIV-1-specific responses and set point at 3 months post infection; **a** Gag, **b** Pol, **c** Nef. **d**–**f** Spearman correlation between the magnitude of HIV-1-specific responses and set point at 1 year post infection; **d** Gag, **e** Pol, **f** Nef. **g**–**i** Spearman correlation between relative magnitude of HIV-1-specific responses and set point at 3 months post infection; **g** Gag, **h** Pol, **i** Nef. **j**–**l** Spearman correlation between relative magnitude of HIV-1-specific responses and set point at 1 year post infection; **j** Gag, **k** Pol, **l** Nef. Relative magnitude was defined in this study as the proportion of magnitude in a specific protein to the total virus-specific magnitude

To clarify the relationship between CTL responses and viral control in the CRF01_AE subtype, we analyzed the effects of magnitude and breadth of Gag-, Pol- and Nef-specific CTL responses at 3 months and 1 year post infection on viral set point. Neither magnitude nor breadth of Gag-, Pol- or Nef-specific responses were correlated with viral set point during the first year of infection (*p* > 0.05, Fig. 2a–f and Additional file 3: Figure S1a–S1f), although the magnitude of Nef at 1 year post infection was significantly positively correlated with viral set point (*p* = 0.01, *r* = 0.642, Fig. 2f).

Several studies have suggested that relative magnitude and breadth, which were defined in this study as the proportion of magnitude and number of reactive peptides in a specific protein to the total virus-specific magnitude and the total number of recognized peptides, respectively, are more sensitive markers of viral control than magnitude and breadth [16, 37]. Thus, we further analyzed the effect of relative magnitude and relative breadth of Gag-, Pol- and Nef-specific CTL responses on viral set point during the first year of infection. We observed a significantly negative correlation between the relative magnitude of Gag and viral set point at 3 months (*p* = 0.002, *r* = −0.726, Fig. 2g) and 1 year (*p* = 0.025, *r* = −0.574, Fig. 2j) post infection. However, no correlation was observed between the relative magnitude of Pol-specific responses and viral set point during the first year of infection (*p* > 0.05, Fig. 2h–k). In contrast, the relative magnitude of Nef at 1 year post infection was significantly positively correlated with viral set point (*p* = 0.004, *r* = 0.697, Fig. 2l). In addition, no correlation was observed between the relative breadth of Gag-, Pol- and Nef-specific responses and viral set point at 3 months or 1 year post infection (Additional file 3: Figure S1g-S1l). This suggests that the higher relative magnitude of Gag presented during the first year of infection leads to improved viral control.

### Multi-layered Gag immunodominant responses were correlated with viral control during the first year of infection

**Fig. 3:**
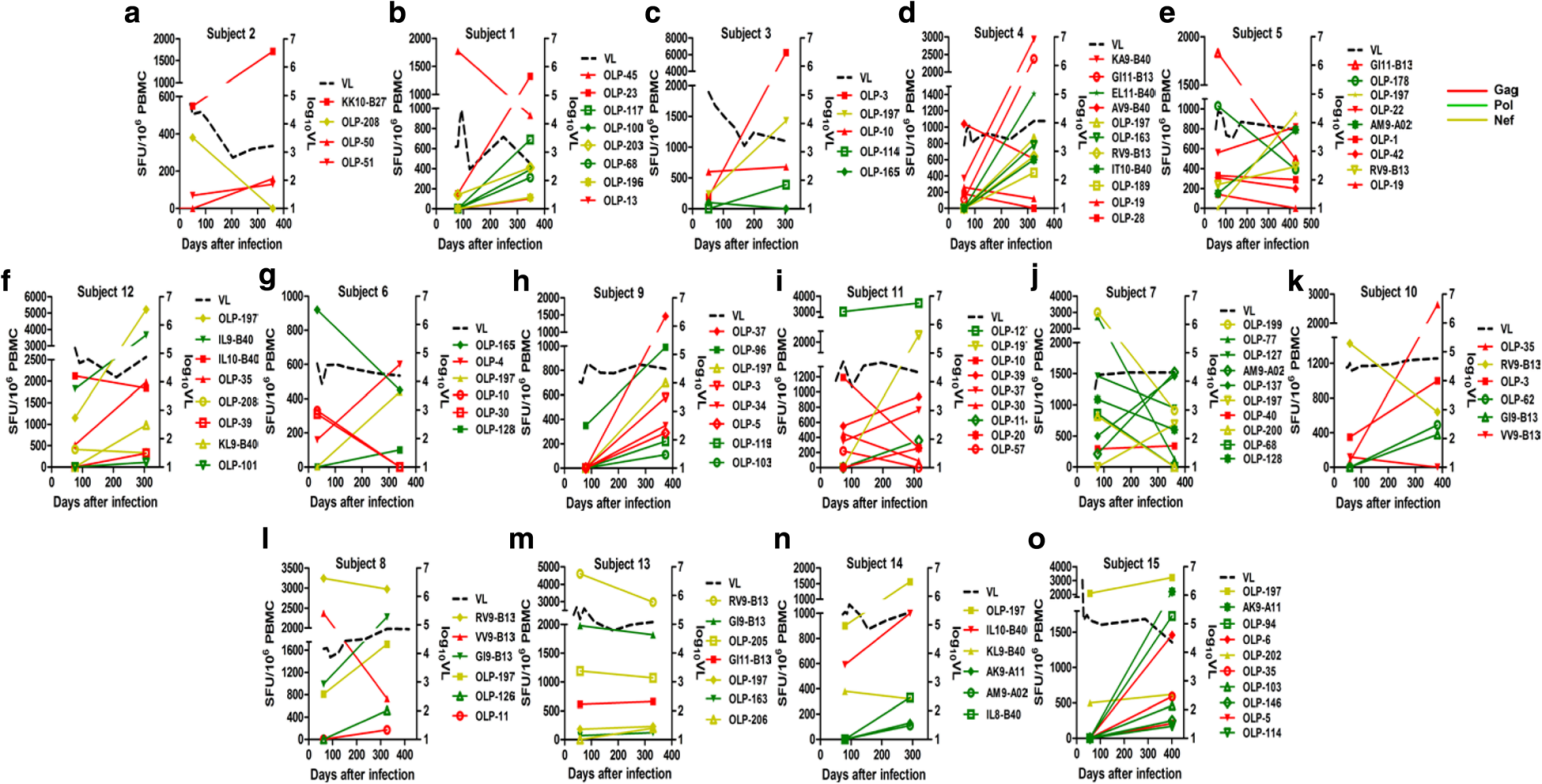
HIV-1-specific CTL kinetics in different subjects. **a** Subject 2, **b** Subject 1, **c** Subject 3, **d** Subject 4, **e** Subject 5, **f** Subject 12, **g** Subject 6, **h** Subject 9, **i** Subject 11, **j** Subject 7, **k** Subject 10, **l** Subject 8, **m** Subject 13, **n** Subject 14, **o** Subject 15. HIV-1-specific CD8^+^ T-cell against 18 mer overlapping peptides and optimal epitopes are shown as solid lines. Viral loads are shown as dotted lines. Epitopes containing OLPs (overlapping peptides) indicates that it was an 18 mer overlapping peptide, and containing HLA type indicates that it was an optimal epitope restricted by a certain HLA type. Position and sequence of the optimal epitopes see Additional file 2: Table S2. Colored lines represent different protein responses in each subject. Red, green and yellow lines represent Gag, Pol and Nef responses, respectively

A higher relative magnitude of Gag is likely associated with improved viral control, indicating an immunodominant response, which is defined as one of the strongest responses for each subject at each time point in this study, targeting the Gag protein may also be related to better viral control. We analyzed the relationship between immunodominant response and viral set point for each subject to clarify the effect of Gag immunodominant response on viral control. Different subjects possessed shifting immunodominance hierarchies over time due to interactions between host and virus. Based on different characteristics of immunodominance and viral set point, we classified the 15 subjects into 3 groups. Group 1 was characterized by having multi-layered immunodominant responses targeting Gag during the first year of infection, which was defined as having Gag immunodominant responses targeting the same epitope or different epitopes at both 3 month and 1 year post infection to force persistent immune pressure on virus. 5 subjects belonging to group 1 showed lower viral set points, ranging from 2.84 to 3.75 log_10_copies/ml. Of the 5 subjects, subject 2 had an immunodominant response targeting the KK10 epitope restricted by HLA-B27 at 3 months and 1 year post infection. However, the other 4 subjects (subject 1, 3, 4 and 5) had immunodominant responses targeting different Gag epitopes at 3 months. The immunodominance hierarchies subsequently shifted over time, and the subjects developed another layer of immunodominant responses targeting new Gag epitopes at later stages of infection (Fig. 3a–e). Group 2 was characterized by having immunodominant responses targeting different proteins during the first year of infection, the viral set points of the 6 subjects belonging to group 2 ranged from 4.29 to 4.62 log_10_copies/ml. Of the 6 subjects, subject 12 had Gag immunodominant responses at 3 months, but not at 1 year post infection (Fig. 3f). Subject 6, 9 and 11 had Pol immunodominant responses at 3 months post infection, and subject 11 had a persistent Pol immunodominant response during the first year of infection (Fig. 3g–i). Subject 7 and 10 had Nef immunodominant responses at 3 months, but not at 1 year post infection (Fig. 3j, k). Group 3 was characterized by having persistent immunodominant responses targeting Nef during the first year of infection, 4 subjects belonging to group 3 showed the highest viral set points among these three groups, ranging from 4.43 to 5.38 log_10_copies/ml (Fig. 3l–o). None of the subjects in group 2 nor group 3 had multi-layered immunodominant responses targeting Gag during the first year of infection.

We further compared the viral set points from different subjects according to Gag immunodominance and found that the viral set point in group 1 was significantly lower than in group 2 and group 3 (3.33 log_10_copies/ml VS 4.69 log_10_copies/ml, *p* = 0.002), meaning that the viral set points in subjects who had multi-layered immunodominant responses targeting Gag were significantly lower than in subjects without the same response during the first year of infection. This suggests that multi-layered immunodominant responses targeting Gag during the first year of infection were correlated with viral control.

### Gag immunodominant responses exerted pressure on the virus during early infection

The observation that multi-layered Gag-specific immunodominant responses during the first year of infection were correlated with viral control suggested that Gag immunodominant responses may exert strong immune pressure on the virus. To study epitope mutation under the pressure of Gag immunodominant responses, we analyzed Gag cloning sequences during the first year of infection. Epitope and flanking region mutations were defined as amino acid mutations with a ≥50 % change in cloning sequences at 1 year compared to the cloning sequences at 3 months. We observed a large proportion of mutated Gag immunodominant epitopes and their flanking regions over time. The AV9 epitope in subject 4 and the IL10 epitope in subject 12 had mutations at 1 year post infection. In addition, the GI11 epitope in subject 5 had flanking region mutations at 1 year post infection. OLP-45 in subject 1 and OLP-10 in subject 3 had flanking region mutations at 1 year post infection based on putative epitopes within the 2 overlapping peptides. Only the KK10 epitope in subject 2 had no mutations and maintained an immunodominant response at 1 year post infection (Table 2). Epitope and flanking region mutations could hamper CTL responses [6, 38]. With epitope and flanking region mutations, the magnitude of AV9, GI11, OLP-45 and IL10 epitopes were all reduced at 1 year post infection, and only OLP-10 in subject 3 was slightly increased (Fig. 3). Under CTL pressure, 5 out of 6 Gag immunodominant epitopes or their flanking regions mutated over time, suggesting that Gag immunodominant responses at 3 months exerted strong pressure on the virus.

**Table 2:**
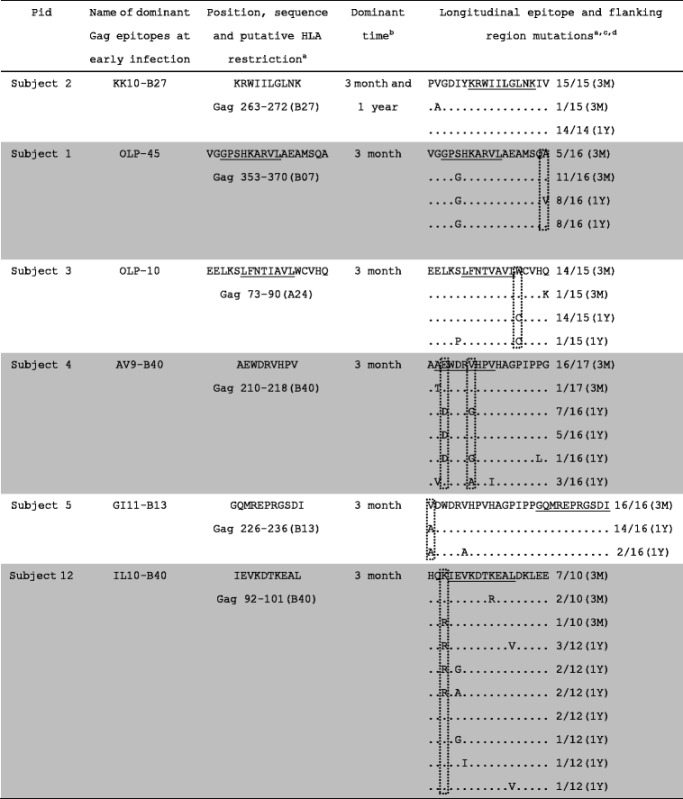
Longitudinal sequence analysis of Gag immunodominant epitopes at 3 months post infection

^a^ HLA types in parentheses denote the putative restricting HLA. Underlined sections denote putative epitopes

^b^Dominant time indicates that the epitope had an immunodominant response at a specific time point

^c^Amino acids in the dashed boxes denote mutation sites. 3M = 3 months, 1Y = 1 year

^d^To specify the flanking region mutation in the GI11 epitope in subject 5, we only show the N terminal amino acid sequence

## Discussion

Evidence has suggested that early CTL responses to HIV-1 infection are vital to viral control [8, 39]. Therefore, a comprehensive understanding of effective CTL response parameters during early infection are important for vaccine design and therapeutic strategy [2]. Through longitudinal analysis of 15 Chinese MSM subjects infected with the CRF01_AE subtype, which is dominant among Chinese MSM subjects and has shown a dramatic uptrend in recent years [32–34], we clarified the characteristics of early CTL responses in CRF01_AE using unique cluster-specific peptides and found that multi-layered immunodominant responses targeting Gag during the first year of infection were significantly associated with improved viral control. This information is useful in vaccine design targeting Chinese MSM subjects infected with the CRF01_AE subtype.

In this study, Gag and Nef proteins were the main targets of CTL responses during the first year of HIV-1 infection, and this was evident from the data after adjusting for the length of amino acids by dividing the amino acids number of the corresponding protein and multiplying by 100 (Fig. 1). These results were similar to the cohort study regarding the B subtype in Streeck et al. [40], although differed from the cohort study in the C subtype in Radebe et al. [41] in which the breadth of Pol was broader at 3 months post infection. This discordance may be due to differences in viral subtype and HLA distribution.

It was generally believed that the viral set point was established about 4 months post infection [42]. CTL responses at 3 months post infection were detected to find host response contribute to viral set point. Moreover, CTL responses at 1 year post infection were also detected to illustrate the CTL responses dynamics. Through correlation analysis between CTL responses and viral set points, we found that the relative magnitude of Gag was significantly negatively correlated with viral set point in CRF01_AE during the first year of infection (Fig. 2), suggesting that the higher proportion of Gag magnitude was associated with improved viral control. This is similar to the results presented in Masemola et al. [16] and Zuniga et al. [37]. Through longitudinal analysis of Gag immunodominant responses and viral set points in each subject, we found that multi-layered immunodominant responses targeting Gag during the first year of infection were correlated with viral control (Fig. 3). In addition, we noticed that subject 12, who had a relatively high viral set point (4.62 log_10_ copies/ml), had a Gag immunodominant response at 3 months. Under host immune pressure, the immunodominant epitope mutated and magnitude was decreased at 1 year post infection. However, no immunodominant responses targeting Gag developed during later stages of infection (Fig. 3f and Table 2), suggesting that only having an immunodominant Gag response in early infection is not sufficient to control the virus. Two other subjects (subject 9 and 10) with relatively high viral set points (4.49 and 4.50 log_10_copies/ml, respectively) only had Gag immunodominant responses at 1 year post infection, which suggests that developing Gag immunodominant responses in early stages of infection is important for viral control (Fig. 3h, k). These data suggest that multi-layered defenses during the first year of infection are needed to effectively control the virus. Tulk et al. found that Gag immunodominance during early infection was correlated with increased plasma IL-2 and MIP-β levels [14], which are associated with better disease control [43, 44]. This may provide one of the reasons why multi-layered Gag immunodominant responses during the first year of infection were correlated with viral control.

We observed that both the magnitude and relative magnitude of Nef responses at 1 year post infection were significantly positively correlated with viral set point (Fig. 2), which was similar to results from Kiepiela et al. [13]. We also found that the viral set points in 4 subjects (subject 8, 13, 14 and 15) who had persistent immunodominant responses targeting Nef were high (Fig. 3), which was consistent with the study of Radebe et al. [45], suggesting that these responses targeting Nef protein are driven by the level of viremia, rather than being responsible for lowering viral load.

Through longitudinal analysis of Gag cloning sequences during the first year of infection, we found that Gag immunodominant responses at 3 months may exert strong pressure on the virus. Five out of 6 Gag immunodominant epitopes or their flanking regions had mutations at 1 year post infection (Table 2). Moreover, the magnitude of the 5 epitopes with mutations within epitopes or their flanking regions all decreased over time, except for the OLP-10 peptide in subject 3 in which the magnitude slightly increased (Fig. 3 and Table 2). Although we did not validate mutated immunodominant epitopes at 1 year post infection, the decline in magnitude suggests that epitope and flanking region mutations could hinder CTL responses [6, 38]. Only the B27 restricted KK10 epitope in subject 2 had no mutations and still maintained an immunodominant response at 1 year post infection (Fig. 3 and Table 2). Many studies have shown that targeting the KK10 epitope could effectively control the virus [17], because escape mutations in the KK10 epitope could confer a large fitness cost to the virus. Escape mutations did not occur until the development of compensatory mutations restored viral replication in chronic infection [17]. This result was similar to the Liu et al. [11] study, which reported that immunodominant responses within an individual were a key reason for escape mutations. These data suggest that Gag immunodominant responses at 3 months exert strong pressure on the virus, which drives the virus to mutate and escape immune pressure. Several subjects (subject 1, 3, 4 and 5) with improved viral control gained another layer of defense, developing new Gag immunodominant responses at later stages of infection and forcing persistent strong immune pressure on the virus, thereby indicating that multi-layered Gag-specific immunodominant responses during the first year of infection may contribute to better viral control.

In this study, we aimed to explore the protective CTL responses to CRF01_AE correlative with viral control, which were suitable for vaccine design and immunotherapy among Chinese MSM subjects, so the published optimal epitopes restricted by the most common HLA-I alleles in the Chinese population were also selected and tested [35]. Some responses might be missed, but the conclusion was believed not to be affected, because most of the known protective epitopes were located in Gag, Pol and Nef proteins [46], which had been screened with the CRF01_AE cluster II specific overlapping peptides. Additionally, only CRF01_AE cluster II HIV-1 infected cases were included in this study, since predominant proportion (81.3 %) of MSM subjects infected HIV-1 strains belonged to CRF01_AE cluster II in this Liaoning MSM prospective cohort [32]. However, whether the results of this study were also appropriate for CRF01_AE cluster I infected cases need further validation.

## Conclusions

Through longitudinal analysis of CTL responses and Gag cloning sequences from 15 MSM subjects with the CRF01_AE primary infection, we found that multi-layered immunodominant responses targeting Gag during the first year of infection were correlated with viral control. We speculate that combined selection of multiple Gag immunodominant epitopes as candidate immunogens at early stages of infection may force a multi-layered defense on HIV-1 in order to achieve better viral control. These data provide a theoretical basis for vaccine design targeting the CRF01_AE subtype in MSM subjects.

## Additional files

Additional file 1: Table S1. Peptide matrix setup for Gag, Pol and Nef. (XLSX 12 kb) Additional file 2: Table S2. Sequences, position and HLA restriction of optimal epitopes used in this study. (XLSX 24 kb) Additional file 3: Figure S1. Association between viral set point and breadth and relative breadth of HIV-1-specific CD8^+^ T-cell responses. (DOCX 1183 kb)

## Abbreviations

CTL: Cytotoxic T-cell
ELISPOT: Enzyme-linked immunospot
HIV-1: Human immunodeficiency virus type 1
HLA: Human leukocyte antigen
MSM: Men who have sex with men
OLPs: Overlapping peptides
PBMCs: Peripheral blood mononuclear cells
PCR-SSP: Polymerase chain reaction sequence-specified primer
PHA: Phytohemagglutinin
SFCs: Spot-forming cells

## References

[1] McMichael AJ, Rowland-Jones SL (2001). Cellular immune responses to HIV. Nature.

[2] McMichael AJ, Borrow P, Tomaras GD, Goonetilleke N, Haynes BF (2009). The immune response during acute HIV-1 infection: clues for vaccine development. Nat Rev Immunol.

[3] BORROW P, LEWICKI H, HAHN BH, SHAW GM, OLDSTONE MBA (1994). Virus-specific CD8+ cytotoxic T-lymphocyte activity associated with control of viremia in primary human immunodeficiency virus type 1 infection. J Virol.

[4] KOUP RA, SAFRIT JT, CAO Y, ANDREWS CA, McLEOD G, BORKOWSKY W (1994). Temporal association of cellular immune responses with the initial control of viremia in primary human immunodeficiency virus type 1 syndrome. J Virol.

[5] Bernardin F, Kong D, Peddada L, Baxter-Lowe LA, Delwart E (2005). Human immunodeficiency virus mutations during the first month of infection are preferentially found in known cytotoxic T-lymphocyte epitopes. J Virol.

[6] Goonetilleke N, Liu MKP, Salazar-Gonzalez JF, Ferrari G, Giorgi E, Ganusov VV (2009). The first T cell response to transmitted/founder virus contributes to the control of acute viremia in HIV-1 infection. J Exp Med.

[7] Kloverpris HN, Harndahl M, Leslie AJ, Carlson JM, Ismail N, van der Stok M (2012). HIV control through a single nucleotide on the HLA-B locus. J Virol.

[8] Streeck H, Nixon DF (2010). T cell immunity in acute HIV-1 infection. J Infect Dis.

[9] Streeck H, Jolin JS, Qi Y, Yassine-Diab B, Johnson RC, Kwon DS (2009). Human Immunodeficiency Virus Type 1-Specific CD8+ T-Cell Responses during Primary Infection Are Major Determinants of the Viral Set Point and Loss of CD4+ T Cells. J Virol.

[10] Mellors JW, Munoz A, Giorgi JV, Margolick JB, Tassoni CJ, Gupta P (1997). Plasma viral load and CD4+ lymphocytes as prognostic markers of HIV-1 infection. Ann Intern Med.

[11] Liu MK, Hawkins N, Ritchie AJ, Ganusov VV, Whale V, Brackenridge S (2013). Vertical T cell immunodominance and epitope entropy determine HIV-1 escape. J Clin Invest.

[12] Leslie AJ, Pfafferott KJ, Chetty P, Draenert R, Addo MM, Feeney M (2004). HIV evolution: CTL escape mutation and reversion after transmission. Nat Med.

[13] Kiepiela P, Ngumbela K, Thobakgale C, Ramduth D, Honeyborne I, Moodley E (2007). CD8+ T-cell responses to different HIV proteins have discordant associations with viral load. Nat Med.

[14] Turk G, Ghiglione Y, Falivene J, Socias ME, Laufer N, Coloccini RS (2013). Early Gag immunodominance of the HIV-specific T-cell response during acute/early infection is associated with higher CD8+ T-cell antiviral activity and correlates with preservation of the CD4+ T-cell compartment. J Virol.

[15] Saez-Cirion A, Sinet M, Shin SY, Urrutia A, Versmisse P, Lacabaratz C (2009). Heterogeneity in HIV suppression by CD8 T cells from HIV controllers: association with Gag-specific CD8 T cell responses. J Immunol.

[16] Masemola A, Mashishi T, Khoury G, Mohube P, Mokgotho P, Vardas E (2004). Hierarchical targeting of subtype C human immunodeficiency virus type 1 proteins by CD8+ T cells: correlation with viral load. J Virol.

[17] Goulder PJ, Phillips RE, Colbert RA, McAdam S, Ogg G, Nowak MA (1997). Late escape from an immunodominant cytotoxic T-lymphocyte response associated with progression to AIDS. Nat Med.

[18] Geldmacher C, Currier JR, Herrmann E, Haule A, Kuta E, McCutchan F (2007). CD8 T-cell recognition of multiple epitopes within specific Gag regions is associated with maintenance of a low steady-state viremia in human immunodeficiency virus type 1-seropositive patients. J Virol.

[19] Goulder PJ, Watkins DI (2008). Impact of MHC class I diversity on immune control of immunodeficiency virus replication. Nat Rev Immunol.

[20] Kiepiela P, Leslie AJ, Honeyborne I, Ramduth D, Thobakgale C, Chetty S (2004). Dominant influence of HLA-B in mediating the potential co-evolution of HIV and HLA. Nature.

[21] Gao X, Nelson GW, Peter Karacki BA, Martin MP, Phair J, Kaslow R (2001). Effect of a single amino acid change in MHC Class I molecules on the rate of progression to AIDS. N Engl J Med.

[22] Frahm N, Kiepiela P, Adams S, Linde CH, Hewitt HS, Sango K (2005). Control of human immunodeficiency virus replication by cytotoxic T lymphocytes targeting subdominant epitopes. Nat Immunol.

[23] Matthews PC, Koyanagi M, Kloverpris HN, Harndahl M, Stryhn A, Akahoshi T (2012). Differential Clade-Specific HLA-B*3501 Association with HIV-1 Disease Outcome Is Linked to Immunogenicity of a Single Gag Epitope. J Virol.

[24] Lichterfeld M, Yu XG, Le Gall S, Altfeld M (2005). Immunodominance of HIV-1-specific CD8(+) T-cell responses in acute HIV-1 infection: at the crossroads of viral and host genetics. Trends Immunol.

[25] Yu XG, Addo MM, Rosenberg ES, Rodriguez WR, Lee PK, Fitzpatrick CA (2002). Consistent patterns in the development and immunodominance of human immunodeficiency virus type 1 (HIV-1)-specific CD8+ T-cell responses following acute HIV-1 infection. J Virol.

[26] Altfeld M, Kalife ET, Qi Y, Streeck H, Lichterfeld M, Johnston MN (2006). HLA Alleles Associated with Delayed Progression to AIDS Contribute Strongly to the Initial CD8(+) T Cell Response against HIV-1. PLoS Med.

[27] Yewdell JW, Bennink JR (1999). Immunodominance in major histocompatibility complex class I-restricted T lymphocyte responses. Annu Rev Immunol.

[28] Yewdell JW (2006). Confronting complexity: real-world immunodominance in antiviral CD8+ T cell responses. Immunity.

[29] Oseroff C, Peters B, Pasquetto V, Moutaftsi M, Sidney J, Panchanathan V (2008). Dissociation between epitope hierarchy and immunoprevalence in CD8 responses to vaccinia virus western reserve. J Immunol.

[30] Turnbull EL, Wong M, Wang S, Wei X, Jones NA, Conrod KE (2009). Kinetics of expansion of epitope-specific T cell responses during primary HIV-1 infection. J Immunol.

[31] Huang MB, Ye L, Liang BY, Ning CY, Roth WW, Jiang JJ et al. Characterizing the HIV/AIDS Epidemic in the United States and China. Int J Environ Res Public Health. 2015;13(1): doi: 10.3390/ijerph13010030. 10.3390/ijerph13010030 PMC4730421 26703667

[32] An M, Han X, Xu J, Chu Z, Jia M, Wu H (2012). Reconstituting the epidemic history of HIV strain CRF01_AE among men who have sex with men (MSM) in Liaoning, northeastern China: implications for the expanding epidemic among MSM in China. J Virol.

[33] He X, Xing H, Ruan Y, Hong K, Cheng C, Hu Y (2012). A comprehensive mapping of HIV-1 genotypes in various risk groups and regions across china based on a nationwide molecular epidemiologic survey. PLoS One.

[34] Han X, An M, Zhang M, Zhao B, Wu H (2013). Identification of 3 Distinct HIV-1 founding strains responsible for expanding epidemic among men who have sex with men in 9 Chinese cities. J Acquir Immune Defic Syndr.

[35] Gonzalez-Galarza FF, Christmas S, Middleton D, Jones AR (2011). Allele frequency net: a database and online repository for immune gene frequencies in worldwide populations. Nucleic Acids Res.

[36] Zhang H, Han X, Zhao B, An M, Wang Z, Jiang F (2015). Multi-layered HIV-1 gag-specific T cell responses contribute to slow progression in HLA-A*30-B*13-C*06-positive patients. AIDS.

[37] Zuniga R, Lucchetti A, Galvan P, Sanchez S, Sanchez C, Hernandez A (2006). Relative dominance of Gag p24-Specific Cytotoxic T lymphocytes is associated with human immunodeficiency virus control. J Virol.

[38] Ranasinghe SR, Kramer HB, Wright C, Kessler BM, di Gleria K, Zhang Y (2011). The antiviral efficacy of HIV-specific CD8(+) T-cells to a conserved epitope is heavily dependent on the infecting HIV-1 isolate. PLoS Pathog.

[39] Brumme ZL, Brumme CJ, Carlson J, Streeck H, John M, Eichbaum Q (2008). Marked epitope- and allele-specific differences in rates of mutation in human immunodeficiency type 1 (HIV-1) Gag, Pol, and Nef cytotoxic T-lymphocyte epitopes in acute/early HIV-1 infection. J Virol.

[40] Streeck H, Lichterfeld M, Alter G, Meier A, Teigen N, Yassine-Diab B (2007). Recognition of a defined region within p24 Gag by CD8+ T cells during primary human immunodeficiency virus type 1 infection in individuals expressing protective HLA class I alleles. J Virol.

[41] Radebe M, Gounder K, Mokgoro M, Ndhlovu ZM, Mncube Z, Mkhize L (2015). Broad and persistent Gag-specific CD8+ T-cell responses are associated with viral control but rarely drive viral escape during primary HIV-1 infection. AIDS.

[42] Schacker TW, Hughes JP, Shea T, Coombs RW, Corey L (1998). Biological and virologic characteristics of primary HIV infection. Ann Intern Med.

[43] Harari A, Petitpierre S, Vallelian F, Pantaleo G (2004). Skewed representation of functionally distinct populations of virus-specific CD4 T cells in HIV-1-infected subjects with progressive disease: changes after antiretroviral therapy. Blood.

[44] Thobakgale CF, Streeck H, Mkhwanazi N, Mncube Z, Maphumulo L, Chonco F (2011). Short communication: CD8(+) T cell polyfunctionality profiles in progressive and nonprogressive pediatric HIV type 1 infection. AIDS Res Hum Retroviruses.

[45] Radebe M, Nair K, Chonco F, Bishop K, Wright JK, van der Stok M (2011). Limited immunogenicity of HIV CD8+ T-Cell Epitopes in acute Clade C virus infection. J Infect Dis.

[46] HIV Databases. Los Alamos National Laboratory, Los Alamos. 2016. http://www.hiv.lanl.gov/content/immunology. Accessed 18 July 2016

