# Supplementary material for: Multi-layered Gag-specific immunodominant responses contribute to improved viral control in the CRF01_AE subtype of HIV-1-infected MSM subjects
Source: BMC Immunol. 2016 Aug 30;17(1):28. doi: 10.1186/s12865-016-0166-8 (PMC5006414; doi:10.1186/s12865-016-0166-8)
Supplement: Additional file 3: Figure S1. — Association between viral set point and breadth and relative breadth of HIV-1-specific CD8+ T-cell responses. (DOCX 1183 kb) [file 12865_2016_166_MOESM3_ESM.docx]

**b**

**c**

**a**









**f**

**e**

**d**









setpoint

(Log_10_ copies/ml)

Breadth of responses

**i**

**h**

**g**









**l**

**k**

**j**









Relative breadth of responses

**Figure S1.** Association between viral set point and breadth and relative breadth of HIV-1-specific CD8^+^ T-cell responses. (a-c) Spearman correlation between breadth of HIV-1-specific responses and set point at 3 months post infection; (a) Gag, (b) Pol, (c) Nef. (d-f) Spearman correlation between the breadth of HIV-1-specific responses and set point at 1 year post infection; (d) Gag, (e) Pol, (f) Nef. (g-i) Spearman correlation between relative breadth of HIV-1-specific responses and set point at 3 months post infection; (g) Gag, (h) Pol, (i) Nef. (j-l) Spearman correlation between relative breadth of HIV-1-specific responses and set point at 1 year post infection; (j) Gag, (k) Pol, (l) Nef. Relative breadth was defined in this study as the proportion of number of reactive peptides in a specific protein to the total number of recognized peptides
